# Evaluating the Financial Factors Influencing Maternal, Newborn, and Child Health in Africa: Tobit Regression and Data Envelopment Analysis

**DOI:** 10.2196/59703

**Published:** 2025-11-28

**Authors:** Youssef Er-Rays, Meriem M'dioud, Hamid Ait-Lemqeddem, Badreddine El Moutaqi

**Affiliations:** 1 Polydisciplinary Faculty of Larache, Abdelmalek Essaadi University, Tetouan, Morocco, 212 5399-79316; 2 École nationale des sciences appliquées, Ibn Tofail University, Kenitra, Morocco

**Keywords:** financial determinants, maternal, newborn, and child health, health care efficiency, Africa, health expenditure, data envelopment analysis, Tobit regression

## Abstract

**Background:**

Despite international efforts, maternal, newborn, and child health (MNCH) outcomes in Africa continue to lag due to inefficient health systems and underperforming financial frameworks. Financial factors—such as total health expenditure, health coverage indices, and spending per capita—are key but understudied drivers of MNCH service efficiency.

**Objective:**

This study investigates the extent to which financial inputs influence the technical efficiency of MNCH service delivery across 46 African countries. The aim is to generate evidence for health financing policies that can enhance both efficiency and health equity.

**Methods:**

We adopted a 2-stage analytical framework. First, data envelopment analysis using a variable returns-to-scale, input-oriented model was applied to measure technical efficiency. Second, Tobit regression identified the financial determinants of inefficiency. Explanatory variables included current health expenditures, a health coverage index, and current health expenditures per capita.

**Results:**

Only 12 of 46 countries (26%) achieved full technical efficiency (efficiency score=1), while the rest (n=34, 74%) were inefficient, with a mean score of 0.849. Efficiency was notably lower in low-income countries (mean 0.810) compared to upper-middle-income countries (mean 0.940). Tobit regression showed that increased current health expenditure significantly reduced inefficiency (*β*=–.0811; *P*=.001). Conversely, a higher health coverage index unexpectedly increased inefficiency (*β*=.0155; *P*=.001), suggesting that expanded coverage without improved governance or resource capacity may strain systems. Health expenditure per capita was not statistically significant. Model 2 demonstrated stronger explanatory power (pseudo *R*²=0.8943).

**Conclusions:**

Financial factors, particularly total health expenditure, play a decisive role in shaping MNCH efficiency across African nations. However, expanding health coverage without parallel improvements in system governance may exacerbate inefficiencies. To enhance MNCH outcomes, policy efforts must focus on increasing and strategically allocating financial resources while strengthening institutional accountability and performance.

## Introduction

Maternal, newborn, and child health (MNCH) is a crucial aspect of global well-being, as outlined in Sustainable Development Goals (SDGs) 3.1 and 3.2. Despite global efforts, Africa continues to face the highest MNCH mortality rates, with 287,000 women dying in 2020 and 5 million children dying in 2021. The World Health Organization (WHO) highlights significant regional imbalances in MNCH outcomes across Africa [1]. A WHO report predicts a slowdown in Africa’s progress against maternal and infant mortality over the past decade [2]. The Atlas of African Health Statistics 2022 reveals that increased investment is needed to accelerate progress toward the SDG on health. Maternal mortality is one of the most difficult targets to achieve, with an estimated 390 women dying in childbirth for every 100,000 live births by 2030 [2]. To reach the SDG target, Africa needs an 86% reduction from 2017 rates, which is unrealistic at the current rate of decline. The region’s infant mortality rate stands at 72 per 1000 live births, with an expected 54 deaths per 1000 live births by 2030 [2]. Although Africa has made significant progress in some areas, such as vaccine coverage, the slowdown has been exacerbated by the COVID-19 pandemic disrupting crucial health services and a resurgence in vaccine-preventable disease outbreaks. Inadequate investment in health and funding for health programs are major drawbacks to meeting the SDG on health. These persistent challenges are exacerbated by weak leadership, corruption, and systemic weaknesses within African health systems, leading to profound inequalities despite overall global mortality halving by 2021 [3-5].

Addressing this critical MNCH challenge requires a deep understanding of how health care resources are used. Therefore, investigating the efficiency of health care systems is paramount to identifying areas where resource allocation can be optimized to improve health outcomes, especially in contexts with limited resources, like many African countries. Global health care system evaluations are essential for pinpointing such improvement areas, and recent research consistently focuses on health care performance [6-11].

Data envelopment analysis (DEA) models are widely recognized and used as tools for evaluating health care efficiency. These models effectively assess the performance of decision-making units (DMUs) by measuring their ability to generate multiple outputs from multiple inputs [12,13]. Research using DEA to compare health care systems has been extensive across various regions. For instance, studies have explored efficiency in 34 Organisation for Economic Co-operation and Development member nations [14], 30 European states [15], 20 Arab countries [16], the Middle East and North Africa region [17], 18 nations within the Middle East and North Africa region [18], and 46 Asian countries [19]. A recurring theme in these studies is the consistent use of variables such as health spending, the number of doctors, and the number of hospital beds as inputs, while life expectancy and infant mortality rates frequently serve as key outcome measures. This methodological consistency, despite the diversity of regions and specific approaches, significantly contributes to a comprehensive understanding of health care system efficiency on a global scale.

However, despite the global prevalence of DEA in health care efficiency studies, research specifically focusing on health system efficiency in Africa remains limited. Musoke et al [20] noted a relative drop in DEA research on the continent, although this approach has seen increased adoption in African studies over the last decade. This gap highlights a critical need for more region-specific analysis to understand and address the unique efficiency challenges within African health systems, particularly in the context of improving MNCH outcomes.

In 2023, Musoke et al [20] compared the health systems of 29 of the least developed African countries. The inputs included domestic general government health, domestic private health, external health, and out-of-pocket health. Outputs included the under-5 survival rate, maternal survival ratio, life expectancy at birth, and infant survival rate.

Top et al [21] examined 36 African health care systems, considering health expenditures in the gross domestic product; medical professionals, nurses, and bed capacity per 1000 individuals; the unemployment rate; and the Gini coefficient. Life expectancy at birth and 1/(infant mortality rate) were the study’s output variables.

Two studies assessed the effectiveness of health care systems in 45 African countries using infant mortality rates and per capita health expenditure and real gross domestic product [22,23]. Another study found that health care infrastructure in sub-Saharan African countries is ineffective due to management weaknesses at multiple levels [24]. Kirigia et al [25,26] investigated efficiency using factors like per capita total health expenditure, adult literacy rate, and male and female life expectancies as outcome variables [25-27].

In a separate study, Arhin et al [28] assessed the ability of the health system to achieve the universal health coverage (UHC) goal by drawing evidence from 30 African countries. The study integrated per capita health spending and physician and hospital data as inputs, with the UHC index serving as the output metric.

However, Qu et al [29] undertook a comparative analysis encompassing 49 African countries from 2000 to 2017. They introduced an innovative methodology that amalgamates DEA with the Gini coefficient to assess the efficacy of technology inequality in addressing environmental issues.

Recent studies have evaluated the efficiency of maternal and child health services using DEA, particularly in developing and middle-income countries such as Morocco. One study, by Youssef et al, used a 2-stage DEA model on 76 MNCH primary health care units in Morocco, revealing an average efficiency score of 0.779 under constant returns to scale (CRS). The study also found significant disparities across provinces, with Boujdour ranking the lowest. Tobit regression revealed that rural health dispensaries and support programs for high-risk pregnancies positively influenced efficiency [9]. A longitudinal study by the same authors used a longitudinal dataset covering 9 years, applying both input- and output-oriented DEA, the Malmquist index, and Tobit regression to assess hospital performance. The results showed an average input-oriented efficiency score of 0.76 and an output-oriented score of 0.23, with mixed productivity trends [11].

Several studies have used DEA to assess MNCH efficiency. Other studies explored efficiency through alternative methods, such as a virtual reality tool that reduced pediatric magnetic resonance imaging anesthesia costs [30], a parental training program that shortened neonatal intensive care unit stays [31], and a mobile health app that lowered asthma hospitalization expenses. These scalable interventions optimized MNCH budgets by reducing resource consumption.

Comprehensive studies focusing specifically on MNCH across African countries—or even within individual nations—remain scarce, with the notable exception of work by Er-Rays and colleagues [32]. This underscores the originality of this paper, which conducts a novel analysis evaluating the financial determinants influencing MNCH in Africa through the application of DEA and Tobit regression.

The literature reviews an assessment of health care system efficiency in other regions, pointing out that it requires a careful selection of inputs, outputs, and explanatory variables. Most of the studies used inputs, which included health care expenditures, health care personnel (doctors, nurses, midwives), hospital beds, and health facilities. The frequently used outputs consisted of life expectancy, health care utilization, and health outcomes. The most used explanatory variables included financial factors, governance, geographic location, infrastructure, and technology. However, most of these studies neglected to consider the maternal mortality rate, stillbirth rate, neonatal mortality rate, and number of births attended by skilled health personnel. Hence, this original paper addresses the technical efficiency of MNCH in Africa.

Motivated by the imperative to achieve SDGs 3.1 and 3.2 by 2030, it is paramount to assess the effectiveness of health systems in Africa, emphasizing the critical need for Africans to strengthen health system resilience. This research contributes significantly by offering information on adopting best practices from more productive health systems, enriching knowledge about productivity in resource-constrained settings, and presenting valuable literature for future researchers. The paper’s originality lies in the meticulous selection of optimal and explanatory combinations, facilitating an assessment of the technical efficiency of 46 health care systems in Africa using DEA and Tobit regression.

The aim of this study is to evaluate the technical efficiency of MNCH services across 46 African countries using a 2-stage methodology that combines DEA and Tobit regression. Specifically, this research investigates how various health system inputs and contextual explanatory variables affect the performance of MNCH services. We hypothesize that inefficiencies in MNCH services are significantly associated with health expenditures, health workforce availability, corruption, and broader socioeconomic indicators such as income inequality and out-of-pocket health costs.

The subsequent sections detail the structured literature review, methods, results, discussion, conclusions, recommendations, limitations, and future research.

## Methods

### Data Sources and Variables

This study included the latest data from the Global Health Observatory and WHO for 46 African countries, including information between 2005 and 2021 [5].

We selected the input, output, and explanatory variables to evaluate the accuracy of the WHO[5] statistics in describing the efficiency of MNCH. Five inputs and outputs are considered to estimate technical efficiency (Table 1).

**Table 1.** Input, output, and explanatory variables.

Variable	Description	Justification	SDG^a^ link
Inputs			
Hospital beds	Hospital beds per 10,000 population	This measure indicates the capacity of the health infrastructure to provide inpatient MNCH^b^ services, which is crucial for safe deliveries and emergency care.	SDG 3.c.1 (Health workforce and infrastructure)
Medical doctors	Medical doctors per 10,000 population	The availability of skilled personnel for diagnosis and treatment is crucial in reducing maternal and child mortality.	SDG 3.c.1 (Health workforce)
Nursing and midwifery personnel	Nursing and midwifery personnel per 10,000 population	Frontline providers for prenatal, delivery, and postnatal care are crucial for improving MNCH outcomes in low-resource settings.	SDG 3.c.1 (Health workforce)
Outputs			
Neonatal mortality rate	Per 1000 live births (2021)	Measures deaths within the first 28 days, indicating the effectiveness of newborn health interventions.	SDG 3.2 (Neonatal and child mortality)
Stillbirth rate	Per 1000 total births (2021)	Reflects the quality of prenatal and delivery care, highlighting gaps in maternal health services.	SDG 3.2 (Neonatal and child mortality)
Infant mortality rate	Probability of dying between birth and age 1 per 1000 live births	The infant mortality rate serves as a broad indicator of child health, reflecting factors such as vaccination, nutrition, and the effectiveness of early care.	SDG 3.2 (Neonatal and child mortality)
Births attended by skilled health personnel	Percentage	Measures access to quality maternal care, reducing risks during delivery for mothers and newborns.	SDG 3.1 (Maternal mortality)
Maternal mortality ratio	Per 100,000 live births (2020)	The maternal mortality ratio reflects the quality of maternal health services by indicating deaths from pregnancy-related causes.	SDG 3.1 (Maternal mortality)
Proportion of vaccination cards seen	Percentage	This percentage indicates the coverage and monitoring of childhood vaccinations, thereby preventing child mortality from vaccine-preventable diseases.	SDG 3.b (Access to vaccines and medicines)
Explanatory variables			
Current health expenditure	Per capita in US $ (2020)	Measures total health spending per person, reflecting investment in MNCH services like vaccinations and skilled birth attendance.	SDG 3.c (Health financing)
External health expenditure	Per capita in US $ (2021)	Captures donor funding for health, supporting MNCH programs in resource-constrained African countries.	SDG 3.c (Health financing)
Proportion of vaccination cards seen	Percentage	This indicates the coverage and monitoring of childhood vaccinations, thereby preventing child mortality from vaccine-preventable diseases.	SDG 3.b (Access to vaccines and medicines)
Composite coverage index	Reproductive, maternal, newborn, and child health interventions, percentage	A composite measure of reproductive, maternal, newborn, and child health intervention coverage (eg, antenatal care, vaccinations) that summarizes health system performance.	SDG 3 (Health and well-being)

a SDG: Sustainable Development Goal.

b MNCH: maternal, newborn, and child health.

### First Stage: DEA

This study used DEA to assess the technical efficiency of health care systems across 46 African countries in delivering MNCH services in the first stage.

Technical efficiency is typically measured using two methods: parametric and nonparametric [8,32-36]. A stochastic frontier production function based on a collection of explanatory variables is used in the parametric approach. The nonparametric technique, on the other hand, uses linear programming to assess the relative efficiency of DMUs by generating an ideal mix of inputs and outputs based on the best-performing unit in the collection [33,37].

Farrel introduced the DEA method [38], and Charnes et al [39] and Banker et al [40] further developed this method. The most common technique is DEA, which may be used independently or in conjunction with a secondary analysis involving the Malmquist index [41], Tobit regression [42], and correlation efficiency. Traditionally, two models are used to calculate the DEA: the CCR model developed by Charnes, Cooper, and Rhodes [39] based on the assumption of CRS and the BCC model proposed by Banker, Charnes, and Cooper based on the assumption of variable returns to scale (VRS) [40]. In the CRS model, outputs are assumed to increase proportionally with inputs, meaning that there are no economies or diseconomies of scale. This simplifies comparisons between similar-sized DMUs [39,40]. In contrast, the VRS model allows for economies and diseconomies of scale, recognizing that each DMU may have an optimal operating size. This model is better suited for comparing DMUs of different sizes [40] as it isolates pure technical efficiency from the influence of scale. DEA models can be categorized as either input-oriented or output-oriented, depending on the relationship between inputs and outputs.

DEA is a widely used method for assessing the relative efficiency of DMUs [33,37-41,43,44]. It is particularly useful when there are multiple inputs and outputs involved in the evaluation process. DEA provides a framework for DMUs and those that achieve the highest level of output given a set of inputs [12,13]. In this study, the CRS and VRS were oriented [45,46].


$$\begin{array}{rl} \text{Max}\;\frac{\sum_{r=1}^{s}u_{rk}y_{rj}+u_{0}}{\sum_{i=1}^{m}v_{ik}x_{ik}}\\ \text{contraints; Max}\; & \frac{\sum_{r=1}^{s}u_{rk}y_{rj}+u_{0}}{\sum_{i=1}^{m}v_{ik}x_{ik}}\leq 1,\;(j,1,2,3,\ldots ,n)\\ v_{rk},\;v_{ik}\geq \varepsilon §gt;0,\;(r=1,2,\ldots ,s),\;(i=1,2,\ldots ,m),\;u_{0}\in R \end{array}$$

The definitions and explanations of these variables are presented as follows:

- *x_ik_*: input *i* used by DMU *k*
- *y_rj_*: output *r* produced by DMU *j*
- *v_ki_*: weight (or multiplier) assigned to input *i* for DMU *k*
- *u_rk_*: weight (or multiplier) assigned to output *r* for DMU *k*
- *u₀*: a constant term (often used in affine DEA models, possibly capturing returns to scale or environmental influences)

The definitions and explanations of these indices are presented as follows:

- *m*: total number of inputs
- *s*: total number of outputs
- *n*: total number of DMUs being evaluated
- *j:* index for DMUs, where *j=1, 2, ..., n*
- *i*: index for inputs, where *i=1, 2, ..., m*
- *r:* index for outputs, where *r=1, 2, ..., s*

The following formula shows the input-oriented VRS model, with results obtained using DEAP (version 2.1) [47] in previous studies [21,40].

By comparing inefficient countries against the efficiency frontier (formed by the most efficient peers), DEA identified countries with the potential for efficiency improvement. The efficiency scores generated in this first stage were then used as a dependent variable in the Tobit regression model to analyze the influence of financial factors on inefficiency.

### Second Stage: Tobit Model

To explore the financial determinants of inefficiency in MNCH service delivery, the second stage used Tobit regression, suitable for censored dependent variables—here, the DEA efficiency scores were bounded between 0 and 1.

The Tobit model was used to analyze the determinants of inefficiency in health care service delivery, specifically regarding MNCH in African countries. The Tobit model is appropriate when the dependent variable is censored, meaning some values are unobserved beyond a certain threshold [42,48]. In this case, the dependent variable is the efficiency score, bounded between 0 and 1 and censored at 0.

Data were first prepared in Microsoft Excel (Microsoft Corp) and then imported into STATA 18 (StataCorp LLC) for statistical analysis. The standard Tobit model is expressed as [42,48]:


$$yi*=x_{i}^{\prime }\beta +u{\;}_{i}(i=1,\ldots ,n)\;\;u_{i}\left\{ \begin{array}{ll} y_{i}*,, & \text{if }yi*§gt;0\\ 0, & \text{if }yi*\leq 0 \end{array}\right.\;\;u_{i}∼IIN(0,\sigma^{-2})$$

In the formula, there is a latent random variable that is observed as *y* if it is positive and is otherwise observed as equal to zero and the parameter vector *β*∈*R*^k^. The error *I* is a normal independent with a mean of zero and precision of σ^2^>0.

We specified two Tobit regression models.

Model 1 includes the following variables: number of medical doctors (MD), number of nurses and midwives (NM), hospital bed density (HBP), current health care expenditure (CHE), and combined health care expenditure and corruption (CHEC) index.

Model 2 expands upon Model 1 by incorporating variables such as out-of-pocket costs, perceived vaccine access (PVACC), comprehensive country index (CCI), and external health contributions (EXHC).

### Ethical Considerations

Our research did not require formal institutional review board or research ethics board approval as it was based entirely on secondary analysis of publicly available, anonymized data that contained no identifiable personal information and did not involve any direct interaction with human participants.

## Results

Following the methodological approach outlined in the previous section, we first normalized and prepared the dataset of 46 African countries (2005‐2021), using DEAP (version 2.1) to compute technical efficiency scores based on both CRS and VRS input-oriented DEA models. Inputs (HBP, MD, NM) and outputs (neonatal mortality rate, stillbirth rate, infant mortality rate from birth to age 1, births attended by skilled health personnel, maternal mortality per live births, PVACC) were used to estimate efficiency scores for each country. Countries achieving a score of 1 were deemed fully efficient, while scores below 1 indicated relative inefficiency. The analysis produced a frontier of best-performing countries, and inefficient countries were benchmarked against this frontier. These DEA results formed the basis for further analysis using the Tobit model to examine determinants of inefficiency.

### Descriptive Statistics of the Variables Used

Mean values for the key variables to analyze descriptive statistics include 12.1 for HBP (beds per 1000 population), 3.5 for MD, and 15.2 for NM as inputs and 23.5 for neonatal mortality rate, 18.7 for stillbirth rate, 58.43 for under-5 mortality rate, and 41.5 for infant mortality rate from birth to age 1 as outputs. Explanatory variables include 75.6 for births attended by skilled health personnel, 354.2 for maternal mortality per live births, 5.7 for CHE, 134.8 for CHEC, 17.2 for EXHC, 35.3 for out-of-pocket costs, 67.7 for PVACC, and 49.1 for CCI.

### First Stage: DEA

Efficiency scores in DEA range from 0 to 1, with a score of 1 signifying that a DMU, specifically a country, is operating at the peak of efficiency, using the fewest possible inputs to achieve the observed outputs. Scores below 1 indicate relative inefficiency.

Following data preparation and input-output selection as described in the Methods, DEA was conducted using DEAP (version 2.1), applying both CRS and VRS input-oriented models. The DEA models identified which countries were efficient and how far inefficient countries were from the efficiency frontier.

**Figure 1.**
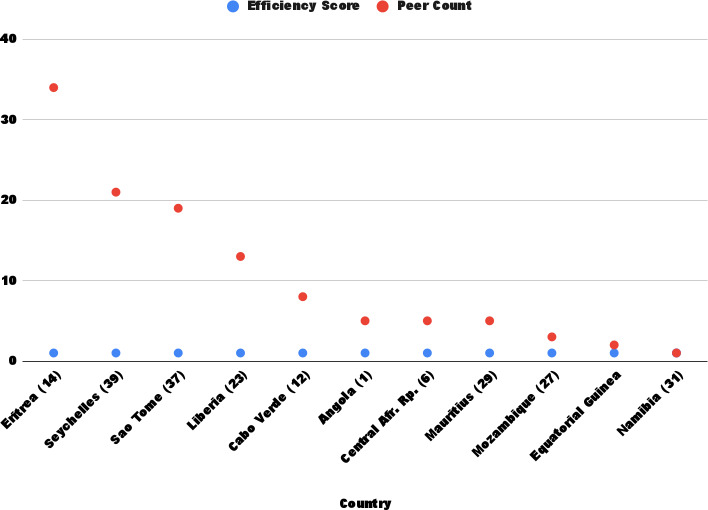
Input-oriented variable returns-to-scale efficiency scores. Blue represents the efficiency scores and red represents the peer count.

Under the VRS model, 12 countries (26%) achieved full efficiency (score=1). The remaining 34 countries (74%) had efficiency scores below 1. The average technical efficiency score (technical efficiency VRS) was 0.849, indicating that, on average, countries could reduce inputs by 15.1% without compromising maternal and child health outcomes (Figure 1).

Gambia showed the lowest performance, with a technical efficiency VRS score of 0.403, whereas Eritrea (DMU 14) was the most frequently referenced efficient benchmark country, serving as a peer for 34 other countries. Seychelles and São Tomé also emerged as key reference points.

**Figure 2.**
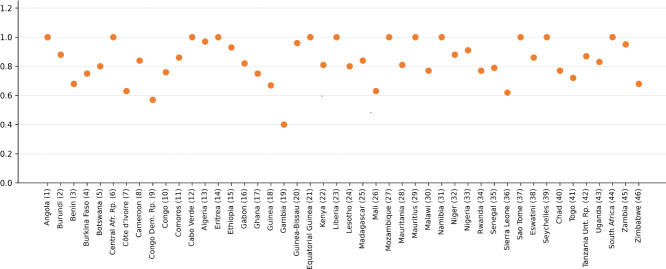
Peer count.

Additionally, the average efficiency score (technical efficiency VRS) across all countries was 0.849 for VRS, signifying that health care systems across the African continent must minimize their inputs by 15% (7 of 46 DMU) under an input orientation. Moreover, the analyzed nations displayed comparable outputs; those identified as efficient used relatively fewer resources than their inefficient counterparts. Eritrea (DMU 14) emerged as the most frequently referenced efficient country, being mentioned 34 times. From this perspective, Eritrea shares similarities with the inefficient countries in the input and output variables considered in this study. Seychelles (referenced 39 times) and São Tomé (referenced 37 times) were the next most referenced efficient countries (refer to Figure 2).

Based on World Bank income classifications, high-income countries exhibited the highest levels of technical efficiency. These nations had the highest average efficiency scores, despite making up only 13% of the sample (6 of 46 DMUs; Table 2). Countries in the middle-income category followed, with an average score of 0.86, representing 39% of the sample (18 of 46 DMUs). In contrast, countries in the lowest income classification had an average efficiency score of 0.810, comprising 43% of the total sample (16 of 46 DMUs). These results suggest that, while income level is a factor in efficiency, some lower-income countries still achieved relatively high performance due to optimal resource utilization.

**Table 2.** World Bank income classification.

World Bank income classification	Average technical efficiency	Decision-making units
Low-income (22 states)	0.810	2, 4, 6, 9, 11, 14, 15, 18, 19, 20, 23, 25, 26, 30, 32, 34, 36, 40, 41, 43, 45
Low-middle income (18 states)	0.860	1, 3, 7, 8, 10, 12, 13, 17, 22, 24, 27, 28, 33, 35, 37, 38, 42, 44, 46
High-upper-middle income (6 states)	0.940	5, 16, 21, 29, 31, 39

### Second Stage: Tobit Model

Table 3 presents the results of the Tobit regressions for both models. The analysis identifies significant predictors of health care inefficiency across African countries with respect to MNCH. In Model 1, CHE was negatively associated with inefficiency scores (*β*=–0.0638*,* SE 0.0217; *t*=–2.94; *P=*.005), indicating that increased expenditure is linked to lower inefficiency. The combined effect of CHEC showed marginal significance (*β*=–.0040, SE 0.0024; *t*=–1.70; *P*=.096).

**Table 3.** Tobit regression. All variables tested using Tobit regression with robust standard errors.

Variable	Tobit model 1				Tobit model 2			
INF (**Inefficiency**)	Coefficient	SE	*t* test (*df*)	*P>|t|*	Coefficient	SE	*t* test (*df*)	*P>|t|*
Number of nurses and midwives	0.0009	0.0045	0.20 (40)	.84	0.0014	0.0051	0.28 (37)	.78
Number of medical doctors	–0.0068	0.0192	–0.36 (40)	.72	–0.0123	0.0168	–0.73 (37)	.47
Hospital bed density	–0.0049	0.0084	–0.58 (40)	.56	–0.0027	0.0089	–0.31 (37)	.76
Current health care expenditure	–0.0638	0.0217	–2.94 (40)	.01	–0.0811	0.0230	–3.52 (37)	.001
Combined health care expenditure and corruption	–0.0040	0.0024	–1.70 (40)	.10	–0.0019	0.0021	–0.94 (37)	.35
Perceived vaccine access	—^a^	—	—	—	–0.0000	0.0019	–0.02 (37)	.98
Comprehensive country index	—	—	—	—	0.0155	0.0041	3.75	.001
Out-of-pocket costs	—	—	—	—	—	—	—	—
External health contributions	—	—	—	—	–0.0049	0.0042	–1.16 (37)	.25
Constant	0.7136	0.1278	5.59 (40)	<.001	0.0820	0.1909	0.43 (37)	.67
Sigma	0.2684	0.0332	—	—	2.2643	0.0276	—	—
**Likelihood Ratio** *χ*² (*df*)	23.37 (5)	—	—	<.001	39.07 (5)	—	—	<.001
Pseudo *R*²	0.5349	—	—	—	0.8943	—	—	—
Log likelihood	–10.16	—	—	—	–2.31	—	—	—

a Not applicable.

In Model 2, CHE remained a significant predictor (*β*=–.0811, SE 0.0230; *t*_37_=–3.52; *P*=.001), and the CCI was positively and significantly associated with inefficiency (*β*=.0155, SE 0.0041; *t*_37_=3.75; *P=*.001). All other variables, including MD, NM, HBP, PVACC, and EXHC, were not statistically significant (all *P >*.05).

The likelihood ratio *χ*^2^ test was significant in both models (Model 1: *χ²*_5_=23.37, *P<*.001; Model 2: *χ²*_5_=39.07, *P<*.001), indicating that the models explained a substantial portion of the variability in inefficiency scores. Pseudo *R²* values were 0.5349 for Model 1 and 0.8943 for Model 2, suggesting better explanatory power in the second model.

## Discussion

This study assessed the efficiency of MNCH systems in 46 African countries using a 2-stage approach: DEA to measure technical efficiency, followed by a Tobit regression to identify determinants of inefficiency. It found that only 26% of countries were technically efficient, while 74% were inefficient. The study also identified key determinants of inefficiency, such as CHE, corruption in health expenditure, and the CCI. Higher-income countries showed better efficiency, while low-income countries had the lowest average efficiency score. The study also found that corruption in health expenditure had a marginally significant negative association with inefficiency, while the CCI was positively and significantly associated with it. Other variables like the number of medical doctors, the number of hospitals, vaccination coverage, and out-of-pocket costs were not statistically significant.

Health systems aim to ensure equitable public access to health care services and judicious resource distribution. The responsibility for funding these requirements lies with the public. The 2030 SDGs urge governments to adopt reforms to enforce regulations in this realm, as emphasized by SDG 3. Most MNCH services rely on health care resources, and the SDGs emphasize the need for efficient funding. This study analyzed the efficiency of MNCH services in 46 countries in Africa in the context of the SDGs, using the DEA method in the first stage and Tobit regression in the second stage.

The research findings disclose a disconcerting scenario, elucidating a substantial dissonance between the prevailing maternal and child health metrics in Africa and the specified SDGs for the year 2030.

The Atlas of African Health Statistics 2022 reveals that sub-Saharan Africa faces challenges in reducing maternal mortality and infant mortality rates. By 2030, 390 women will die in childbirth for every 100,000 live births, more than 5 times the 2030 SDG target [2]. To meet the target, Africa needs an 86% reduction from 2017 rates, which is unrealistic. The region’s infant mortality rate is 72 per 1000 live births, and at the current rate of decline, 54 deaths per 1000 live births will be expected [2]. There has been some progress in key health objectives: vaccine coverage has increased, under-5 mortality has fallen by 35%, neonatal death rates dropped by 21%, and maternal mortality declined by 28% [2]. However, the region still has a long way to go, with the COVID-19 pandemic disrupting vital health services and the resurgence of vaccine-preventable disease outbreaks. Inadequate investment in health and funding for health programs are major drawbacks to meeting the SDG on health. Accelerating the agenda to meet its reduction goal will be crucial for reducing under-5 mortality to fewer than 25 deaths per 1000 live births [2].

This alarming disparity between the observed metrics and the established SDG targets underscores the considerable distance that African countries currently find themselves from realizing the objectives outlined in SDG 3. Addressing this discrepancy necessitates a comprehensive evaluation of the efficiency and various influencing variables within the MNCH domain.

The findings from the DEA analysis revealed notably low or medium efficiency for most African countries. This suggests that 22 of 46 states represent low-income countries, followed by 18 of 46 states classified as low-medium income. Eritrea was the most-referenced country. According to the Tobit model analysis, financial factors such as CHE, CCI, and CHEC harmed the inefficiency of the health system related to MNCH. These findings indicated that the health financing system suffers from profound dysfunctions, which hinders the promotion of MNCH in African countries.

According to previous studies on African countries, the performance of health systems was generally low or moderately efficient based on scores [1,20,24,25,27,29]. The WHO reported an average technical efficiency score of 0.79 across its 47 member countries in 2019 [1]. Ibrahim et al [49] assessed health care systems in sub-Saharan Africa and identified them as generally inefficient. During the analyzed period, only three provinces—in Rwanda (2014 and 2015) and in Tanzania (2015)—were found to be efficient.

The study also discovered that governance metrics, notably the rule of law and government efficacy, have a greater impact on health care system efficiency than public health spending. This implies that effective resource management is more important than the amount of money invested in health care systems in sub-Saharan African nations [49]. According to Babalola and Moodley’s findings [50], less than 40% of the facilities tested were efficient. These studies reported parameters such as catchment population, facility ownership, and geography.

Arhin et al [28] discovered that by implementing best practices in instruction, management performance, expenditures on public health, external health funding, and prepayment arrangements, 30 sub-Saharan African health systems can increase UHC levels by 19% while using existing health care resources.

The overall health care efficiency in different African countries is considerable. Notably, Ghana, Sierra Leone, and Burkina Faso all recorded a low technical efficiency score in the provision of MNCH [21,24,49,51,52]. The choices of input and output variables depend on the availability of information in the reports concerning the activities of health establishments in these countries. Technical efficiency varies from one health system to another.

In Ghana’s case, 78% of primary health care institutions have a low efficiency score [53]. Primary health care facilities in KwaZulu-Natal, South Africa, similarly have a 70% low technical efficiency [26]. Alhassan et al [54] found that the geographic location of the centers and their type of ownership were substantially associated with the prediction of efficiency scores rather than the quality of service. Marschall and Flessa’s [51] Tobit model results in Burkina Faso demonstrated that the explanatory variables determining inefficiency in rural health care were highly related to geographical distance and other factors.

The management of African health care systems, particularly in the realm of MNCH, presents a multifaceted challenge encompassing economic, social, political, and infrastructural factors. These challenges include financial constraints, human resource shortages, infrastructure deficiencies, cultural and social barriers, governance issues, high disease burdens, inadequate health facility capacity, suboptimal utilization of health services, leakages, and corruption. Economically advanced countries such as Eritrea, Seychelles, Mauritius, Namibia, South Africa, and São Tomé exhibit efficient health systems. However, economically less developed countries encounter difficulties in providing and accessing health services due to their developmental status and less robust institutional frameworks.

A literature review revealed that countries like Ghana, Sierra Leone, and Burkina Faso all demonstrated low-efficiency scores in delivering MNCH services. It is imperative to advocate for enhanced resource allocation strategies, prioritize efficient utilization of health care resources, optimize infrastructure enhancements, invest in workforce training, and embrace technology to streamline service delivery. Health authorities are urged to consider comprehensive policy reforms aimed at addressing operational inefficiencies identified in the study. These reforms should be strategic and tailored to enhancing the overall effectiveness of health care systems in the domain of maternal and child health.

This study assessed the effectiveness of health care systems; however, its precision relied on data from the WHO, which may have overlooked key determinants influencing MNCH outcomes. Additionally, the study assumed homogeneity in production functions across diverse African countries, potentially oversimplifying variations in health care infrastructure, socioeconomic conditions, and cultural factors. Moreover, the study’s focus on internal factors may have neglected external influences such as political stability and global health crises. Generalizing the findings beyond the studied nations is also risky due to the continent’s heterogeneity and the dynamic nature of its efficiency. Future research could explore sustainable financing solutions for health care systems, addressing structural constraints faced by African states.

## Abbreviations

CCI: comprehensive country index
CHE: current health care expenditure
CHEC: combined health care expenditure and corruption
CRS: constant returns to scale
DEA: data envelopment analysis
DMU: decision-making unit
EXHC: external health contributions
HBP: hospital bed density
MD: number of medical doctors
MNCH: maternal, newborn, and child health
NM: number of nurses and midwives
PVACC: perceived vaccine access
SDG: Sustainable Development Goal
UHC: universal health coverage
VRS: variable returns to scale
WHO: World Health Organization
